# Resveratrol prevents hearing loss and a subregion specific- reduction of serotonin reuptake transporter induced by noise exposure in the central auditory system

**DOI:** 10.3389/fnins.2023.1134153

**Published:** 2023-03-24

**Authors:** Long-Quan Cheng, Fang-Qi Shu, Min Zhang, Yuan-Zhong Kai, Zheng-Quan Tang

**Affiliations:** ^1^School of Life Sciences, Anhui University, Hefei, China; ^2^Key Laboratory of Human Microenvironment and Precision Medicine of Anhui Higher Education Institutes, Anhui University, Hefei, China; ^3^Institute of Artificial Intelligence, Hefei Comprehensive National Science Center, Hefei, China

**Keywords:** noise exposure, resveratrol, serotonin reuptake transporter (SERT), cochlear nucleus, inferior colliculus, auditory cortex

## Abstract

Prolonged or excessive exposure to noise can lead to hearing loss, tinnitus and hypersensitivity to sound. The effects of noise exposure on main excitatory and inhibitory neurotransmitter systems in auditory pathway have been extensively investigated. However, little is known about aberrant changes in neuromodulator systems caused by noise exposure. In the current study, we exposed 2-month-old mice to a narrow band noise at 116 dB SPL for 6 h or sham exposure, assessed auditory brainstem responses as well as examined the expression of serotonin reuptake transporter (SERT) in the cochlear nucleus (CN), inferior colliculus (IC), and primary auditory cortex (Au1) using immunohistochemistry. We found that noise exposure resulted in a significant increase in hearing thresholds at 4, 8, 16, 24, and 32 kHz, as well as led to a significant reduction of SERT in dorsal cochlear nucleus (DCN), dorsal IC (ICd), external IC (ICe), and Au1 layers I-IV. This reduction of SERT in these subregions of central auditory system was partially recovered 15 or 30 days after noise exposure. Furthermore, we examined efficacy of resveratrol (RSV) on hearing loss and loss of SERT induced by noise exposure. The results demonstrated that RSV treatment significantly attenuated threshold shifts of auditory brainstem responses and loss of SERT in DCN, ICd, ICe, and Au1 layers I-IV. These findings show that noise exposure can cause hearing loss and subregion-specific loss of SERT in the central auditory system, and RSV treatment could attenuate noise exposure-induced hearing loss and loss of SERT in central auditory system.

## 1. Introduction

Noise pollution is a major environmental health concern in the world. According to WHO, about 20% of the population in EU countries is exposed to environmental noise. Prolonged or excessive exposure to environmental noise can cause hearing loss, tinnitus, and hypersensitivity to sound. Several mechanisms underlying noise exposure-induced hearing loss have been identified. Some previous studies have shown that long term exposure to high-intensity noise can cause tympanic membrane perforation and loss of hair cells in mice (Rimbaud et al., 1973; Yang et al., 2004), as well as result in a reduction in synapses between hair cells and spiral ganglion neurons (Hori et al., 2009; Boero et al., 2021).

Beyond its direct effects on peripheral auditory system, several lines of evidence have suggested that noise exposure may affect hearing and cognition *via* impairment of neurotransmitter systems in the brain. For example, noise trauma can damage spatial learning and memory, possibly through impairing neurogenesis of hippocampal neurons in mice (Liu et al., 2016). Continuous noise exposure in early developing rats may lead to long-term potentiation (LTP) of postsynaptic potentials in the primary auditory cortex and thalamic cortex. In the critical period of hearing, continuous white noise exposure can change the bidirectional thalamic cortical plasticity of rat auditory cortex (Speechley et al., 2007). Continuous noise exposure may induce the aggregation of Aβ and cognitive impairment in mice (Karem et al., 2021). Moderate noise exposure during the early development of mice increases the density of GABA and somatostatin (SOM) cells in the primary auditory cortex (Au1) and the anterior auditory field (AAF), but decreases the density of perineural network cells in Au1. In adult mice, noise exposure can induce an up-regulation of parvalbumin (PV) cells and perineural network around AAF cell bodies (Reinhard et al., 2019). Some studies have suggested that acoustic trauma may induce 17 differential regional-specific change of molecules, including amino acids, fatty acids, glycolic acids, nucleosides, and organic acids. For example, GABA levels are significantly increased only in Au1, while glutamate levels are significantly increased in cerebellum (He et al., 2017).

In addition, evidence suggest that noise exposure may impact 5-HT system in the CNS. A previous study has shown that chronic broadband white noise exposure can increase extracellular 5-HT concentrations in the rat cerebellum, hypothalamus, pons, and striatum (Ravindran et al., 2005). Some studies also showed that noise exposure can cause the loss of the serotonin reuptake transporter (SERT) in the IC, frontal cortex, hippocampus, striatum, thalamus, hypothalamus, midbrain, and auditory cortex (Kang et al., 2013; Liu et al., 2019). In addition, noise exposure can induce an increase in 5-HT level in the central auditory system (Cransac et al., 1998), and the increase of 5-HT level in the central auditory system may be related to the generation of tinnitus (Simpson and Davies, 2000; Liu et al., 2003), a condition associated with neuronal hyperactivity in the central auditory system (Kaltenbach and Godfrey, 2008; Olthof et al., 2022). SERT plays an important role in regulating clearance of extracellular 5-HT. Interestingly, some previous studies have suggested that SERT is highly expressed in some subregions of IC and Au1 (Papesh and Hurley, 2012; Pan et al., 2021). However, it is unclear whether noise exposure differentially impacts SERT in subregions of auditory system. Therefore, understanding how noise exposure affect the expression of SERT in the central auditory system may thus provide an insight into mechanisms underlying tinnitus.

Resveratrol (3, 5, 4′-trihydroxystilbene, RSV) is a naturally occurring compound with a variety of pharmacological properties, including anti-inflammatory, antioxidant, and neuroprotective effects (Soleas et al., 1997; Hao and He, 2004; Sonmez et al., 2007). Recent studies have suggested that RSV can alleviate noise exposure-induced or age-related hearing loss in mice (Pang et al., 2019; Liu et al., 2022). One of the potential mechanisms by which RSV alleviate hearing loss is protecting against noise exposure-induced hair cells damage or loss (Seidman et al., 2003; Hanci et al., 2016). In addition, RSV has also been demonstrated to exert a neuroprotective effect against SERT loss in central auditory system and non-auditory system (Li et al., 2019). However, it is unclear whether treatment with RSV attenuate the noise exposure-induced change of SERT in different brain regions, particularly in such brain subregions with high SERT expression level.

Therefore, the aim of the current study is to determine whether noise exposure (NE) differently affect SERT in subregions of central auditory system and test the protective effect of RSV against noise exposure-induced impairment of SERT.

## 2. Materials and methods

### 2.1. Animals

**FIGURE 1:**
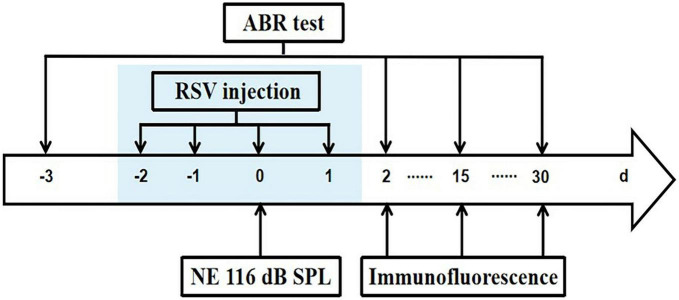
Experimental timeline. Mice were exposed to 8 k 116 dB SPL noise on day 0. Resveratrol (RSV) was injected on –2, –1, 0, and 1 days. Initial ABR tests were performed on 3 days before NE and repeated ABR tests were done 2, 15, and 30 days after NE. After ABR recordings, immunostaining was carried out on 2, 15, and 30 days after NE.

8-week old C57BL/6J male mice were obtained from Biotechnology Co., Ltd. (Beijing) and housed in the facility at least 1 week for acclimatization before any procedures. The facility is controlled for temperature and humidity, has a 12 h light/dark cycle (lights on 8 a.m.). The mice were provided with food and water *ad libitum*. Mice with normal hearing assessed by measuring the thresholds of auditory brainstem response (ABR) were used in the study, and were randomly assigned into 5 groups (Control, Noise Exposure, Noise Exposure + Resveratrol Treatment, 15 and 30 days post Noise Exposure, each group consisted of 7 mice). Mice were sacrificed for immunostaining on day 2, 15, and 30 after noise exposure following ABR test (Figure 1). All animal procedures were approved by the Institutional Animal Care and Use Committee of Anhui University (protocol numbers 2020-039).

### 2.2. Noise exposure

Noise exposures were performed on the mice that were treated with vehicle (DMSO) or resveratrol (RSV, 30 mg/kg, intraperitoneal injection) (NE and NE + RSV, respectively) 2 days before, on the day of noise and 1 day after noise exposure. A total of 15 days post-noise exposure (15dPNE) and 30 days post-exposure (30dPNE) groups were given 15 and 30 days’ recovery after noise exposure. Control group (CTRL) mice did not receive noise exposure.

Mice of NE, NE + RSV, 15dPNE, and 30dPNE four groups were anesthetized *via* intraperitoneal injections of 50 mg/kg pentobarbital sodium and were placed in a soundproof booth (Cosen, Beijing) throughout noise exposure. Mice were exposed to a narrowband noise (bandwidth 100 Hz) centered at 8 kHz, 116 dB sound pressure level (SPL) for 6 h. The acoustic stimuli were generated by Tucker-Davis Technology (TDT; Tucker-Davis Technologies, Alachua, FL, USA) hardware from a JBL GT7-6 speaker which situated 20 cm above the mice. The control mice were anesthetized and placed in the sound-attenuating booth without noise exposure for 6 h same to other groups.

### 2.3. Auditory brainstem response (ABR) test

Hearing thresholds were determined at 4, 8, 16, 24, and 32 kHz using the RZ6 recording system (TDT, Alachua, FL, USA) 3 days before NE and 2, 15, and 30 days after NE (Figure 1). The mice were anesthetized by pentobarbital sodium (50 mg/kg, ip), kept warm with a thermostatic hearing pad in a soundproof booth (Cosen, Beijing) for the recordings. Subdermal needle electrode was inserted at the vertex, the reference electrode was located at below the left ear, and the grounding electrode located at below the right ear. The wide-band click sound (10 ms, 22/s) and tone burst sound (4, 8, 16, 24, and 32 kHz, 0.5 ms rise/fall period, no plateau period, alternating phase) were presented using a free-filed speaker (ES1 electrostatic speaker, TDT). The average response of 1,024 stimuli at each frequency were obtained by reducing the sound intensity from 90 dB to 20 dB in 5 dB decrements. ABR waves I and II were used to determine the ABR thresholds for each frequency. The hearing threshold was determined by identifying the lowest intensity where visually detectable and recognizable peaks appear in the response waveform.

### 2.4. Immunofluorescence

After ABR recordings, the mice were deeply anesthetized with a lethal intraperitoneal injection of sodium pentobarbital and perfused intracardially with 20 ml 0.01 M phosphate-buffered saline (PBS, pH 7.4) followed by 20 ml 4% paraformaldehyde (PFA) in PBS. After perfusion, the brain was removed and fixed in 4% PFA for 12 h. Then the brain was cryoprotected with 15% sucrose in PBS (0.01 M) at 4°C for one day and then changed to a 30% sucrose solution at 4°C for 2 days. The brains containing cochlear nucleus (CN), inferior colliculus (IC), and auditory cortex (Au1), were cut in 40 μm-thick coronal sections using a cryostat microtome (Leica CM 1900, Leica Biosystems, Wetzlar, Germany) and harvested for immunostaining.

We selected three consecutive slices from each of brain regions containing CN, IC, and Au1 of each animal. The sections were washed three times with 0.01 M PBS for 10 min each time, and subsequently permeabilized in 5% bovine serum protein, 0.5% Triton X-100 in PBS for 1 h. After again being washed in 0.01 M PBS three times, brain slices were incubated with SERT antibody (1:1,000, immunostar, USA, 243300) overnight at 4°C. Then the brain slices were washed three times with 0.01 M PBS and incubated with IFKine Green Affinipure Donkey Anti-Rabbit IgG (1:800, Abbkine Scientific, Wuhan, China, A24221) for 2 h at room temperature. Finally, after being washed again in PBS, the slices were mounted on slides and coverslipped with mounting medium. The tissues of the CTRL, NE, and NE + RSV groups were processed at the same time.

Serotonin reuptake transporter-immunoreactive (ir) were acquired using laser-scanning confocal microscopy (Olympus VS200) in 5% laser intensity. 20 × and 40 × confocal images were collected serially throughout the subregions of the cochlear nucleus (CN), inferior colliculus (IC), and primary auditory cortex (Au1), and all parameters were identical when collected images of each brain regions. Based on the previous study (Anderson et al., 2009), we divided primary auditory cortex (Au1) into layers I-IV and layers V-VI, the area from the pia to 50% of the cortical depth was defined as layers I-IV and from 50% depth to the white matter was defined as layer V-VI. The immunofluorescence intensity of each region in three consecutive brain slices was analyzed by Image J (National Institutes of Health, Bethesda, MD, USA).

### 2.5. Data analysis

All data were expressed as the mean + SEM and analyzed using Student’s *t-*tests or one-way analysis of variance (ANOVA) followed by the Turkey HSD test. All statistical analysis and plotting were used by origin (OriginLab, Massachusetts, MA, USA). *p < 0.05; ^**^p < 0.01; ^***^p < 0.001.

## 3. Results

### 3.1. Noise exposure induced temporary hearing loss which can be prevented by resveratrol

**FIGURE 2:**
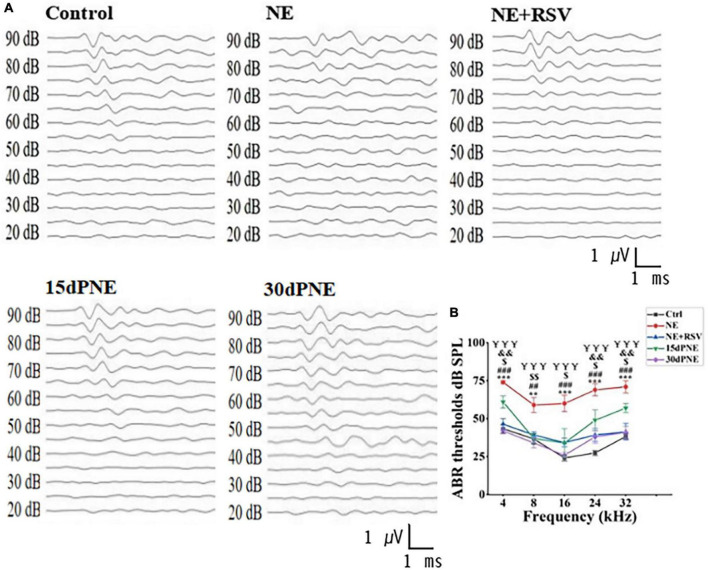
ABR tests show that noise exposure increased reversibly hearing thresholds, and resveratrol treatment prevented hearing thresholds. **(A)** Example waveforms from a control mouse (Ctrl), noise exposure mouse (NE), resveratrol treatment mouse (NE + RSV), a mouse 15 days after noise exposure (15dPNE), and a mouse 30 days following noise exposure (30dPNE). **(B)** The averaged hearing threshold of each group is shown. Compared with the Ctrl group, the hearing threshold of mice in the NE group was significantly increased; the hearing threshold of mice in the NE + RSV group was significantly lower than that of the NE group; improved hearing thresholds were observed in 15dPNE and 30dPNE groups. Values are mean ± SE, data was analyzed by Tukey’s test following one-way of ANOVA. Each group consisted of 7 mice. Statistical difference is represented as *p < 0.05, **p < 0.01, and ***p < 0.001 for comparisons between the control group and NE group; ^#^*p* < 0.05, ^##^*p* < 0.01, and ^###^*p* < 0.001 for the NE + RSV group compared with NE group; $*p* < 0.05, $$*p* < 0.01, and $$$*p* < 0.001 for comparisons between the NE group and 15dPNE group; &*p* < 0.05, &&*p* < 0.01, and &&&*p* < 0.001 for comparisons between the Ctrl group and 15dPNE group; ¥*p* < 0.05, ¥¥*p* < 0.01, and ¥¥¥*p* < 0.001 for comparisons between the NE group and 30dPNE group.

In order to determine how noise exposure affects expression level of SERT in auditory system, we performed ABR test to measure the hearing threshold of mice in different groups: CTRL, NE, NE + RSV, 15dPNE, and 30dPNE. As expected, the mice in NE group exhibit a significant threshold shift (NE group; 74.00 ± 1.00 dB at 4 kHz, 59.00 ± 4.85 dB at 8 kHz, 60.00 ± 5.48 dB at 16 kHz, 69 ± 4.00 dB at 24 kHz, and 71 ± 4.00 dB at 32 kHz), compared with control mice at all frequencies tested (CTRL group; 43.33 ± 2.79 dB at 4 kHz, 36.67 ± 3.33 dB at 8 kHz, 24.17 ± 2.00 dB at 16 kHz, 27.50 ± 1.71 dB at 24 kHz, and 38.33 ± 1.67 dB at 32 kHz: *n* = 7, *p* < 0.01, one-way ANOVA, Figures 2A, B). We further examined the recovery of thresholds in mice exposed to noise. After 15 days noise exposure, the animals exhibited a slight recovery of thresholds. The average of thresholds of all frequencies tested in the 15dPNE group was significantly lower than that in the NE group (NE: 66.60 ± 3.87 dB, 15dPNE: 47.60 ± 5.316 dB; *n* = 7, *p* < 0.01; one-way of ANOVA, Figures 2A, B). Furthermore, the animals exhibited a significant recovery of the averaged threshold of all frequencies tested in the 30dPNE group, compared with that in the NE group (NE: 66.60 ± 3.87 dB, 30dPNE: 36.20 ± 3.05 dB; *n* = 7, *p* < 0.001; one-way of ANOVA, Figures 2A, B), as well as no statistically significant difference in averaged thresholds between the 30dPNE and CTRL groups was detected (CTRL: 34.00 ± 2.30 dB, 30dPNE: 36.20 ± 3.05 dB; *n* = 7, *p* > 0.05; one-way of ANOVA, Figures 2A, B).

We also examined the protective effects of RSV on hearing loss induced by noise exposure and found that the averaged threshold of all frequencies tested in the NE + RSV group was significantly lower than that in the NE group (NE + RSV: 40.15 ± 3.64 dB, NE: 66.60 ± 3.87 dB; *n* = 7, *p* < 0.001; one-way of ANOVA, Figures 2A, B). In addition, there was no significant difference in averaged thresholds between the NE + RSV and CTRL groups (CTRL: 34.00 ± 2.30 dB, NE + RSV: 40.15 ± 3.64 dB; *n* = 7, *p* > 0.05; one-way of ANOVA, Figures 2A, B). These data suggested that noise exposure caused a temporary hearing loss which can be prevented by administration of RSV.

### 3.2. Noise exposure down-regulated SERT in subregion of central auditory system

We next examined SERT fluorescence intensity ratio in central auditory system between CTRL and NE groups, and compared SERT level in subregions of central auditory system, including CN, IC, and Au1. Immunostaining revealed that SERT was highly expressed in the subregions of CN, IC, and Au1. In auditory brain stem, expression level of SERT in the DCN as detected by anti-SERT antibody was significantly higher than that in the VCN (Supplementary Figure 1). In auditory midbrain, expression level of SERT in ICd and ICe was higher than that in the ICc (Supplementary Figure 1). Similarly, SERT was higher expressed in Au1 layers I-IV, compared with that in layers V-VI (Supplementary Figure 1).

**FIGURE 3:**
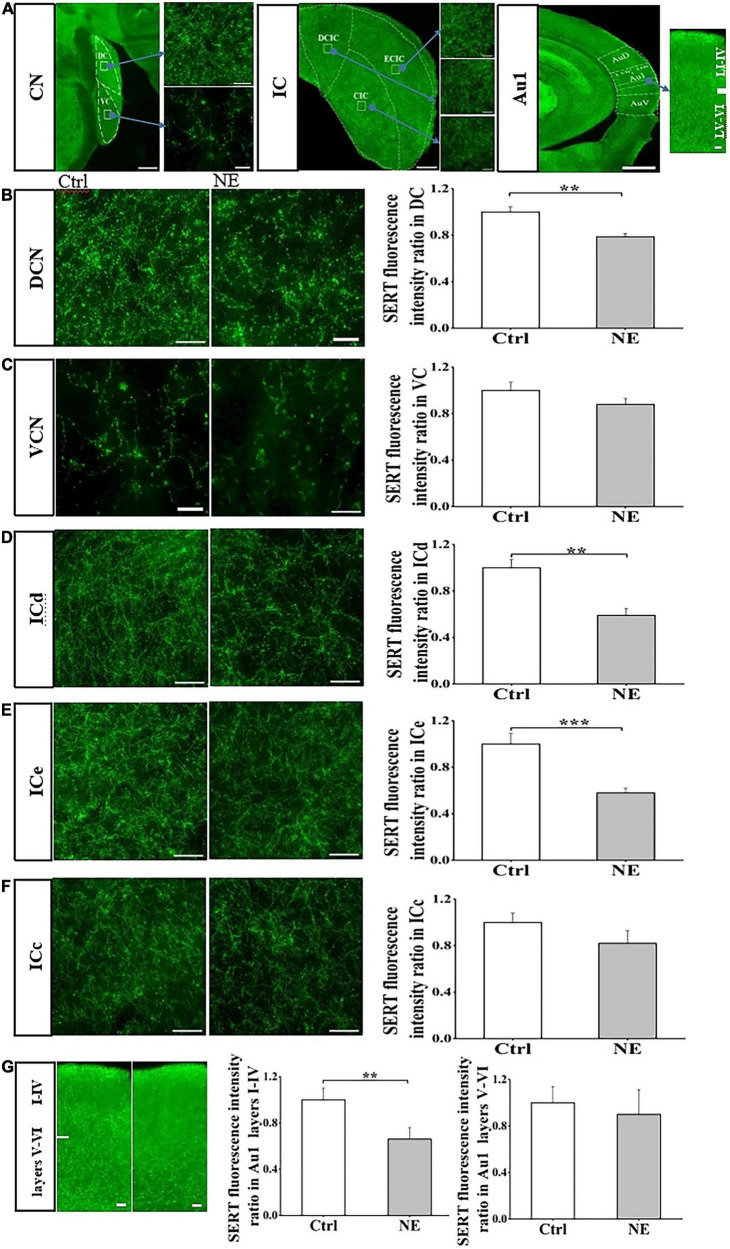
Noise exposure decreased SERT level in auditory brain subregions. **(A)** The photomicrographs taken with a 20 × and 40 × magnification illustrate SERT immunoreactivity in the cochlear nucleus (CN), inferior colliculus (IC), primary auditory cortex (Au1), and their subregions dorsal CN (DCN), ventral CN (VCN), dorsal IC (ICd), external IC (ICe), central (ICc), Au1 layers I-VI. **(B–G)** Left: Micrograph taken with a 40 × magnification show the distribution of SERT^+^ fibers in the DCN, VCN ICd, ICe, ICc, and Au1 layers I-VI. Right: Bar graphs represent the SERT immunofluorescence intensity ratio in these subregions between NE and Ctrl groups. A quantitative immunofluorescence ratio was calculated by dividing SERT immunofluorescence intensity in NE, 15dPNE, 30dPNE groups by that in the Ctrl group. Values are mean ± SE, data was analyzed by Student’s *t*-tests. Ctrl group *n* = 7; NE group *n* = 7. Statistical difference is represented as *p < 0.05, **p < 0.01, ***p < 0.001 for comparisons between the Ctrl group and NE group.

Interestingly, our results showed that SERT expression was significantly down-regulated in DCN, ICd, ICe, and Au1 layers I-IV of the mice in NE group, compared with that in the CTRL group (Figure 3). DCN SERT levels in the mice of NE group was significantly decreased by 21% after noise exposure compared with the control group (*p* < 0.01; Student’s *t-*tests, Figures 3A, B). However, there was no significant difference in VCN SERT levels between the NE group and the CTRL group (*p* > 0.05; Student’s *t-*tests, Figures 3A, C).

We also detected SERT levels of three IC subregions in NE and CTRL groups, and found that ICd and ICe SERT levels of NE group were significantly decreased by 41 and 42% after noise exposure (*p* < 0.01; Student’s *t-*tests, Figures 3A, D–F), without significant change in SERT expression in ICc subregion, compared with the control group (*p* > 0.05; Student’s *t-*tests, Figures 3A, D–F). Similarly, we found that SERT levels of Au1 layers I-IV in NE group was significantly decreased by 34% after noise exposure (*p* < 0.01; Student’s *t-*tests, Figures 3A, G), without significant change in SERT expression in Au1 layers V-VI subregion, compared with the control group (*p* > 0.05; Student’s *t-*tests, Figures 3A, G). Overall, our results clearly showed that the noise exposure caused a decrease in SERT levels in the subregions of CN, IC, and Au1. These data suggested that noise exposure may affect SERT expression in central auditory system in a subregion- and layer-specific manner.

### 3.3. The loss of SERT induced by noise exposure recovered partially 15 and 30 days after noise exposure

**FIGURE 4:**
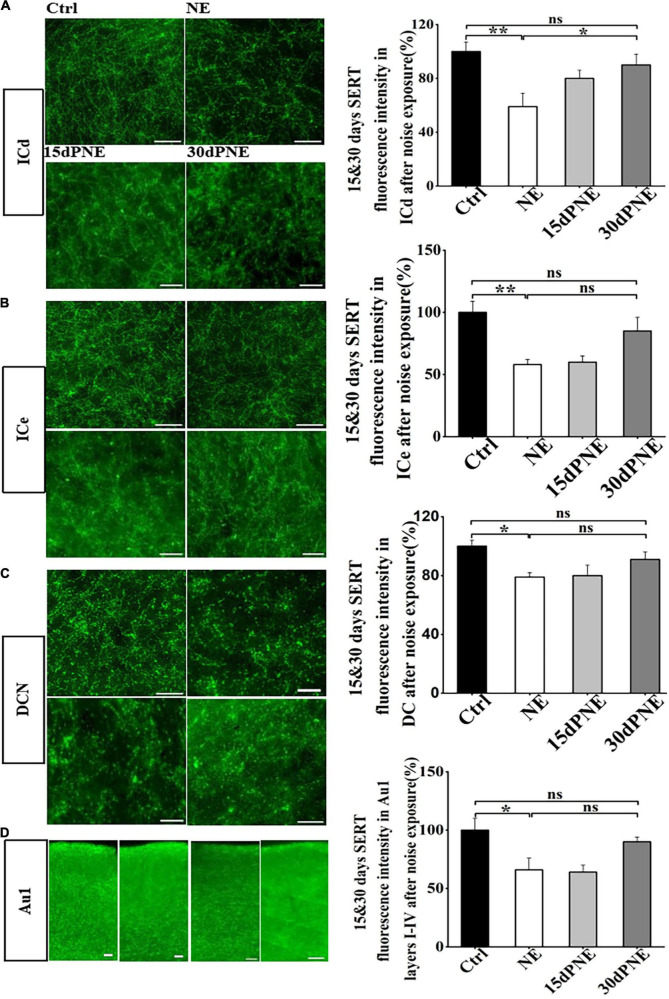
Loss of SERT in some subregions recovered partially 15 and 30 days after noise exposure. **(A–D)** Left: Micrograph taken with a 40 × magnification show the distribution of SERT^+^ fibers in the DCN, ICd, ICe, and Au1 layers I-IV. Right: Bar graphs represent the percentage of the SERT immunofluorescence intensity in these subregions between NE, 15dPNE, 30dPNE groups and the Ctrl group. Values are mean ± SE, data was analyzed by Tukey’s test following one-way ANOVA. Ctrl group *n* = 7; NE group *n* = 7; 15dPNE group *n* = 7; 30dPNE group *n* = 7. Statistical difference is represented as *p < 0.05, **p < 0.01, and ***p < 0.001.

To test whether SERT in subregions of auditory brain areas is reversibly down-regulated by noise exposure, we examined the SERT level after 15 and 30 days after noise exposure. Compared with the mice in NE group, the loss of SERT in ICd in mice of 15dPNE slightly but not significantly recovered (one-way of ANOVA, Figure 4A), but the loss of SERT in ICd in mice of 30dPNE significantly recovered (*p* < 0.05; one way of ANOVA, Figure 4A).

However, we observed that the loss of SERT in DCN, ICe and Au1 layers I-IV in mice of 15dPNE and 30dPNE partially but not significantly recovered (one-way of ANOVA, Figures 4B–D). Furthermore, the results also showed that the loss of SERT in ICc, VCN, and Au1 layers V-VI in mice of 15dPNE and 30dPNE recovered partially (data not shown). Our research showed that noise induced loss of SERT in the subregions of the auditory central system could be partially recovered in a time dependent manner.

### 3.4. Resveratrol prevent the loss of SERT induced by noise exposure

**FIGURE 5:**
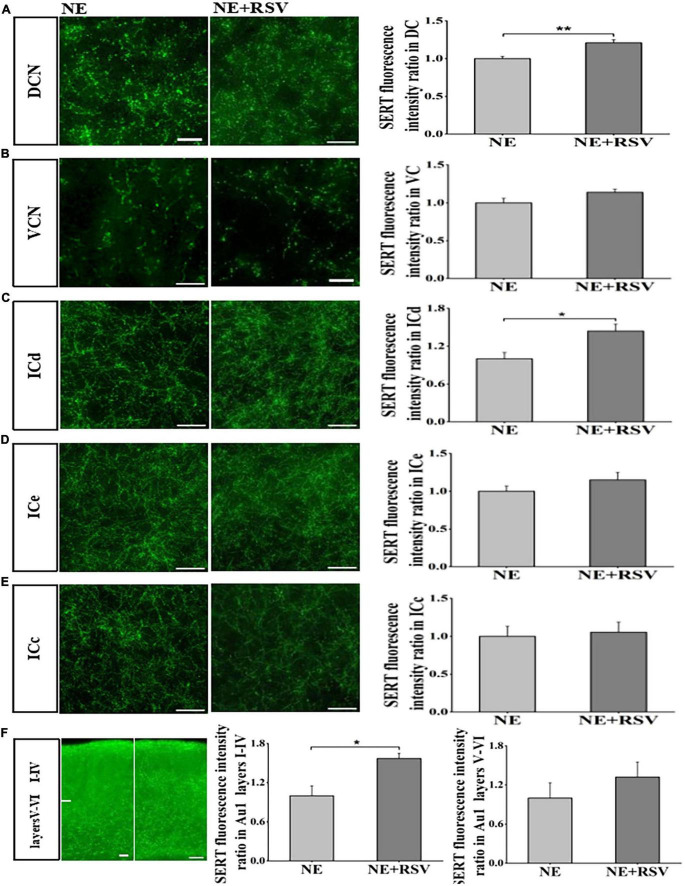
Resveratrol protected against noise-induced SERT loss in auditory brain subregions. **(A–F)** Left: Micrograph taken with a 40 × magnification show the distribution of SERT^+^ fibers in the DCN, VCN ICd, ICe, ICc, and Au1 layers I-VI. Right: Bar graphs represent the SERT immunofluorescence intensity ratio in these subregions between NE + RSV and NE groups. A quantitative immunofluorescence ratio was calculated by dividing SERT immunofluorescence intensity in NE + RSV group by that in the NE group. Values are mean ± SE, data was analyzed by Student’s *t*-tests. NE group *n* = 7; NE + RSV group *n* = 7. Statistical difference is represented as *p < 0.05, **p < 0.01, and ***p< 0.001.

The previous studies suggested that RSV can prevent hearing loss and down-regulation of SERT induced by noise exposure. In our study, we also examined the effects of RSV (30 mg/kg, ip) on noise exposure induced loss of SERT. Using immunostaining, we found that expression levels of SERT in DCN, ICd and Au1 layers I-IV were significantly higher than those in NE mice (*p* < 0.05; *t*-test, Figures 5A, C, F). However, we observed that expression levels of SERT in VCN, ICe, ICc, Au1 layers V-VI partially but not significantly recovered (*p* > 0.05; *t*-test, Figures 5B, D–F). These data suggested that RSV may prevent noise-induced down-regulation of SERT in subregions of auditory brain regions.

## 4. Discussion

In the current study, we reported two major findings. First, we found that noise exposure can induce a temporary hearing loss in mice and a reversible down-regulation of SERT in subregions of central auditory system. Second, administration of RSV can largely prevent noise exposure-induced hearing loss and down-regulation of SERT in central auditory system.

### 4.1. Noise exposure can induce hearing loss and down-regulate SERT in central auditory system

Some previous studies have reported that noise exposure can increase the hearing threshold and cause the loss of hair cells in the cochlea (Shi et al., 2014; Wen et al., 2015; Zhang et al., 2017; Seist et al., 2021; Zheng et al., 2021; Han et al., 2022). Consistently, we also found that noise exposure can cause an increase of hearing threshold in mice. Our present study mainly focused the impact of noise exposure on SERT expression in the central auditory system, thus we did not detect morphological changes in the cochlea of the peripheral auditory system of mice. Previous studies have reported that noise trauma not only induce hearing loss, but also down-regulate the SERT expression in the central auditory system, including IC and Au1 (Li et al., 2019; Liu et al., 2019). Several studies used Small-Animal Positron Emission Tomography (PET) with N, N-dimethyl 1-2–(2-amino-4–[18F] fluorophenylthio) benzylamine (4–[18F]–ADAM, a specific radioactive ligand for SERT) to evaluate SERT changes in the control and noise exposure conditions, and suggest that noise exposure decreases markedly SERT expression in the central auditory system (Kang et al., 2013; Li et al., 2019). In addition, a previous study has shown that noise trauma can down-regulate SERT expression in hippocampus, prefrontal cortex and thalamus (Kang et al., 2013). Our present study showed that SERT was highly expressed in some brain subregions, such as DCN, ICd, ICe, and Au1 layers I-IV. In support, the previous study has suggested that serotonergic fibers differently distribute across subregions of the IC and CN, particularly with higher density in DCN, ICd, and ICe (Hurley and Hall, 2011). Interestingly, noise exposure can induce higher extent loss of SERT particularly in some subregions of auditory brain regions in mice, suggesting that noise exposure affect SERT expression in central auditory system in a subregion-specific manner. While our present studies obtained from central auditory system of mice are generally consistent with those obtained from central auditory system of rat in a previous study (Li et al., 2019), there exists an obvious discrepancy in noise-induced SERT loss between central auditory system of rat and mice. Li et al. (2019) reported that noise exposure induces SERT loss in central auditory system, but they did not observe obvious difference in SERT expression or the effects of noise exposure on SERT in subregions. The most parsimonious explanation for this discrepancy is that SERT are differentially expressed in central auditory system between mice and rat. Our data also showed that noise induced SERT loss in central auditory system partially recovered 15 days after noise exposure, and largely recovered 30 days after noise exposure, suggested that noise induced SERT loss can recover in a time dependent manner.

The main function of SERT in the CNS is to take up 5-HT released by 5-HT neurons. Previous studies had shown that auditory stimulus or restricted movement can increase extracellular 5-HT concentration in the IC, suggesting that the neuromodulation by 5-HT in the central auditory system is related to behavioral states and environmental events (Ruda et al., 1991). In mouse CN, IC, and Au1, we found that SERT was differentially distributed, being mainly observed in DCN, ICe, ICd, and Au1 layers I-IV. Noise exposure largely down-regulated high-level expressions of SERT in these subregions, suggested that noise exposure may affect the functions of these subregions in central auditory system. It has been reported that 5-HT can enhance the excitability of principal cell in the DCN, one of tinnitus generated sites in auditory system (Tang and Trussell, 2015, 2017). Our study indicated that noise exposure can cause the reduction of SERT levels in DCN, which may lead to an increase in extracellular 5-HT and subsequently increase the excitability of the DCN. Previous studies suggest that hyperexcitability of the DCN was associated with tinnitus in animals (Eggermont, 2005; Li et al., 2015). Hearing loss was the largest risk factor for tinnitus, and the risk of tinnitus caused by exposure to high-level sounds in the early life will increase (Figueiredo et al., 2011; Muhr and Rosenhall, 2011; Ryan and Bauer, 2016). Thus, it remains to be explored whether chronic noise exposure promotes structure and biochemical changes in serotonergic circuits in central auditory system, leading to hearing loss and tinnitus-like behavior in animals.

### 4.2. Resveratrol treatment prevents noise exposure-induced hearing loss and the loss of SERT

Several lines of evidence have shown that RSV can alleviate hearing loss induced by noise exposure and prevent the loss of SERT induced by noise exposure (Seidman et al., 2003; Li et al., 2019). In the current study, we examined the effects of RSV on hearing loss and loss of SERT induced by noise exposure, and found that RSV provide protection against noise-induced hearing loss and SERT loss in DCN, ICd, and Au1 layers I-IV subregions, which was consistent with previous reports (Li et al., 2019; Liu et al., 2022). Based on a previous study (Shih et al., 2016), we speculate that RSV may have the potential to protect against noise exposure-induced SERT loss, possibly through binding to SERT. Further study is warranted to confirm this hypothesis.

## 5. Conclusion

In conclusion, our study shows that noise exposure has different effects on subregions SERT levels in the three auditory brain regions. Resveratrol not only has a protective effect on the auditory threshold, but also has a protective effect on the subregions SERT levels of the three auditory brain regions. It can be used as a potential prevention or supplement for the treatment of hearing diseases related to SERT loss.

## Data availability statement

The raw data supporting the conclusions of this article will be made available by the authors, without undue reservation.

## Ethics statement

The animal study was reviewed and approved by the Institutional Animal Care and Use Committee of Anhui University.

## Author contributions

MZ, Y-ZK, and Z-QT conceived and supervised the project, and designed the experiments. L-QC and F-QS performed the experiments, collected data, and analyzed the data. L-QC, MZ, Y-ZK, and Z-QT wrote the manuscript. All authors contributed to the article and approved the submitted version.
