# Supplementary material for: Resveratrol prevents hearing loss and a subregion specific- reduction of serotonin reuptake transporter induced by noise exposure in the central auditory system
Source: Front Neurosci. 2023 Mar 24;17:1134153. doi: 10.3389/fnins.2023.1134153 (PMC10080035; doi:10.3389/fnins.2023.1134153)
Supplement: Supplementary file 1 [file Data_Sheet_1.PDF]

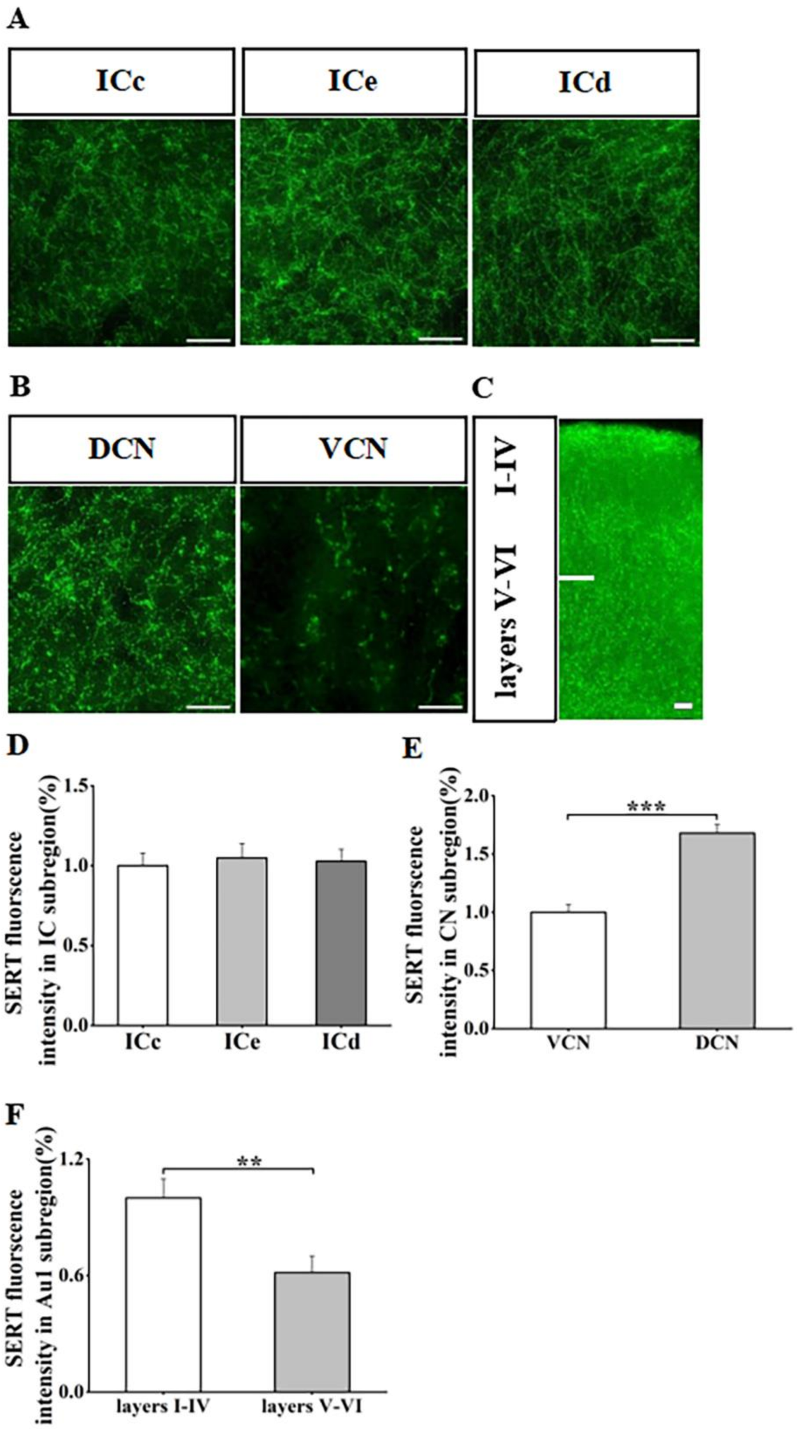

Supplementary Figure S1. **SERT is highly expressed in DCN, ICd, ICe and Au1 layers I-IV.** (**A-C**) Photomicrographs of serotonin reuptake transporter (SERT) by immunofluorescence stain in various brain subregions of the Ctrl group were displayed (n = 7). (**A**) dorsal inferior colliculus (ICd), external inferior colliculus (ICe) and central inferior colliculus (ICc); (**B**) dorsal cochlear nucleus (DCN), venture cochlear nucleus (VCN); (**C**) primary auditory cortex layers I-IV (Au1 layers I-IV), primary auditory

cortex layers V-VI (Au1 layers V-VI). **(D-F)** Column Charts indicated that the ratio of SERT fluorescence intensity of brain subregions in each brain regions. inferior colliculus (IC); cochlear nucleus (CN); primary auditory cortex (Au1). Values are mean ± SE, data was analyzed by Tukey's test following one-way ANOVA and Student's *t* tests. Ctrl group n = 7. Statistical difference is represented as* $p < 0.05$, ** $p < 0.01$, *** $p < 0.001$.
